# Effect of Linewidth on the Relative Intensity Noise in Random Distributed Feedback Raman Fiber Lasers

**DOI:** 10.3390/s22218381

**Published:** 2022-11-01

**Authors:** Sergio Rota-Rodrigo, Daniel Leandro, Giorgio Santarelli, Manuel Lopez-Amo, Juan Diego Ania-Castañón

**Affiliations:** 1 LP2N, Institut d’Optique Graduate School, CNRS, Université de Bordeaux, F-33400 Talence, France; 2 Institute of Smart Cities (ISC), Department of Electrical, Electronic and Communications Engineering, Public University of Navarra (UPNA), Campus de Arrosadia, 31006 Pamplona, Spain; 3 Instituto de Óptica “Daza de Valdés”, IO-CSIC, Serrano 121, 28006 Madrid, Spain

**Keywords:** laser, fiber, distributed feedback, Raman effect, RIN transfer, random distributed feedback fiber lasers

## Abstract

We experimentally explore the relation between spectral linewidth and RIN transfer in half-open cavity random distributed feedback Raman lasers, demonstrating for the first time the possibility of adjusting the pump-to-signal RIN transfer intensity and cut-off frequency by using spectral filtering in the reflector section. We apply this approach to a 50-km laser system, operating in the C-Band, reliant on a standard single-mode fiber. We obtained a minimum bandwidth of 13 pm, which translates into a visible RIN cut-off at 800 MHz.

## 1. Introduction

Random distributed feedback fiber lasers (RDFLs), based on distributed Rayleigh scattering, represent a particular case of random lasing that has been thoroughly investigated in the last decade, given their unique features [1] and broad range of applications. In random distributed feedback lasers, in contrast to the usual localized reflectors present in closed-cavity lasers, the system includes one or more random reflectors in which the light is randomly back-scattered. Because of this, the properties of the random lasers differ significantly from conventional schemes [2]. Several approaches can be employed to provide this random reflection, but in the case of fiber lasers, randomly distributed feedback can be provided by the fiber’s own Rayleigh backscattering. This effect is inherently present in fiber optics, but it is usually weak for short fiber lengths. However, in a laser comprised of a long enough length of a standard low-loss single-mode fiber (usually a few tens of kilometers), and in the presence of distributed gain, it is relatively simple to design a system in which random backscattering dominates over roundtrip reflections [3], leading to random, modeless lasing. Such devices are known as random distributed feedback fiber lasers. Since distributed feedback in low-loss communication fibers is weak, even for spans of tens of kilometers, high amplification ratios are required in order to reach the lasing threshold. Raman amplification is a widespread solution given its distributed nature and the fact that it can be applied to any conventional optical fiber; however, other approaches, such as the use of doped fibers [4], Brillouin amplification [5], or a combination of all of them, also can be used [6] to achieve efficient lasing. Raman-based RDFLs offer stable stimulated emission in a spectral band of few nanometers, but this spectral behavior can be tailored to create multi-wavelength sources with linewidths of few picometers [7]. RDFLs offer many other interesting features, such as broad tunability, high output power and efficiency, multiple open and half-open design possibilities, the ability to be internally modulated without frequency restrictions, and the expected random lasing modeless behavior [8,9]. These properties can be exploited in several applications, such as long-range sensing, where RDFLs have been used as light source for the interrogation of time, wavelength, and coherence division multiplexing networks, at distances of up to 290 km [10]. RDFLs also offer great potential for amplification in long-range communications, although in this regard some key aspects, such as the generation and transmission of relative intensity noise (RIN), have yet to be fully studied. Some of the most relevant studies of RIN transfer in RDFLs [11,12] showed the influence of pump power and fiber characteristics on the frequency-dependent RIN transfer function and shed some light about the differences between RIN transfer in mode-dominated ultra-long lasing and random distributed feedback regimes. In addition, the RIN transfer and noise intensity level are becoming relevant for high-power multiple-order Raman distributed feedback lasers using short fibers [13,14,15,16]. Furthermore, some of this previous work [17] allowed us to theoretically predict that, in some particular configurations, the maximum RIN transfer in RDFLs, which typically happens at low modulation frequencies, could be dampened, leading to an anomalous RIN transfer profile in which pump intensity noise is transferred efficiently only to a mid-frequency band, which could help to reduce noise impairments in telecommunications and sensing. More recently, we demonstrated this effect experimentally on different RDFL configurations [18,19]. In this manuscript, we set out to improve our understanding of RDFLs by experimentally exploring, for the first time, the impact of spectral linewidth control on RIN generation and transfer, as well as the output laser characteristics. The results will prove extremely useful in the design of optimized RDFLs with tunable RIN characteristics, improving their applicability in a variety of areas

## 2. Materials and Methods

**Figure 1:**
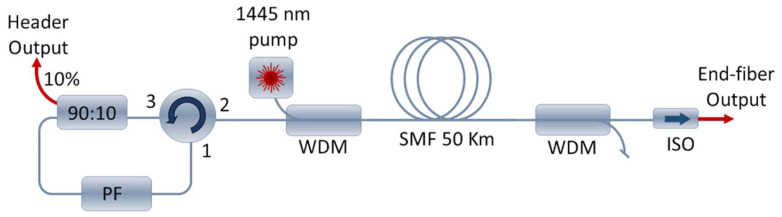
Schematic of the RDFL setup (PF: programmable filter; WDM: wavelength division multiplexer; ISO: isolator).

Our random distributed feedback Raman fiber laser setup is based on a forward-pumped topology (please refer to schematic on Figure 1). The cavity is formed by a 50 km-long single-mode fiber (SMF) in which the Rayleigh backscattering acts as a weak distributed mirror, and a recirculating system based on a circulator and a programmable filter waveshaper (Finisar, WS 1000S), which allows us to control both the RDFL wavelength and linewidth. Two wavelength division multiplexers (WDMs) are located on each side of the 50 km span, one for injecting the pump laser at 1445 nm (IPG Photonics, RLD-5K-1445) and the other for removing the residual pump at the end output. A 90:10 coupler is used for extracting 10% of the signal at the header-output feedback loop. A fiber isolator placed at the end-fiber output is used to avoid reflection.

In order to accurately characterize RIN in our system, we combined low- and high-frequency detection systems. For low frequencies, ranging from 10 Hz to 1 MHz, a low-noise custom-made photodetector (10 MHz bandwidth) and a vector signal analyzer (FFT Agilent 89410A) were used. On the other hand, for high frequencies, a fast photodetector with a 6 GHz bandwidth (DSC 100S) and a spectrum analyzer (R&S FSP30) were employed. Both systems were calibrated and compared, showing an offset of about 2 dB, consistent with both the detection system and the analyzers’ calibration errors. Having more than a one-decade spectrum overlap between the two detection systems allows for offset removing. Finally, an optical spectrum analyzer (OSA, Advantest Q8384) with 0.01 nm resolution was used for monitoring the laser spectra.

## 3. Results and Discussion

**Figure 2:**
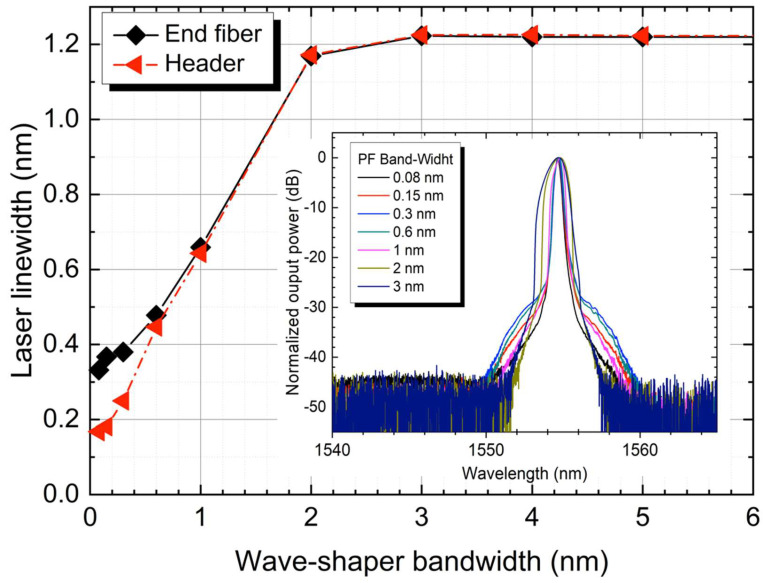
Laser linewidth evolution at end-fiber (black) and header (red), depending on the programmable filter bandwidth selection. (Inset) Spectra at the end-fiber output measured at OSA (resolution 0.01 nm) for different filter BWs.

To determine the relationship between the programmable filter bandwidth (BW) and the actual output linewidth of the RDFL, a characterization was carried out for a pump power of 2 W. Figure 2 shows that the linewidth of the RDFL remains constant around 1.2 nm for filter BWs over 3 nm. This was expected since the characteristic BW of this RDFL without any filtering is about 2.2 nm. Figure 2 (inset) depicts the spectral evolution of the end-fiber output. The broadening effect seen in the base of the spectra can be attributed mainly to nonlinear wave turbulence, as reported before in RDFL [20] and ultra-long Raman fiber lasers [21].

**Figure 3:**
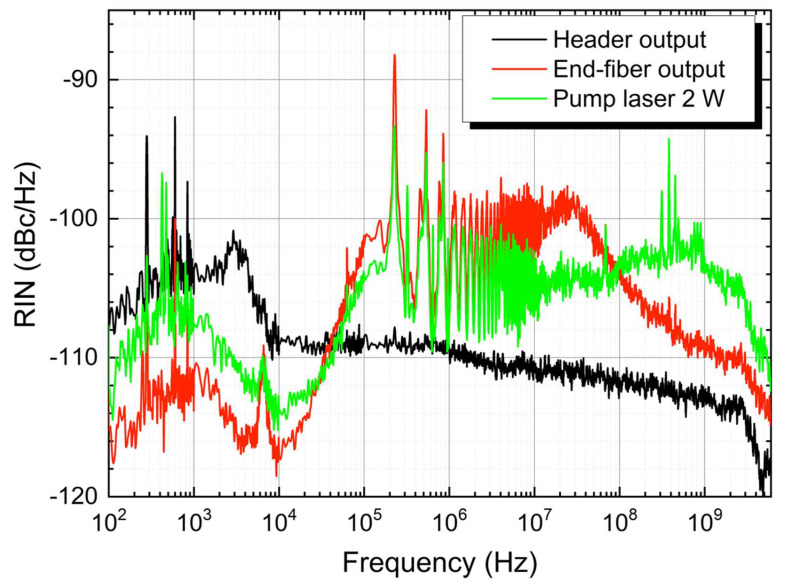
RIN measurements of the RDFL at the end-fiber (red) and header (black) outputs, and the RIN of the pump (green), for a 0.3 nm BW and 2 W pump power.

The measured RIN of the RDFL and the pump, for a 0.3 nm filter BW and 2 W pump power, is depicted in Figure 3. The RDFL output powers were 5 mW and 140 mW at the header and end-fiber outputs, respectively. The noise at the end-fiber output is higher than at the header for frequencies above 40 kHz, due to the additional pump noise transfer taking place in the co-propagated configuration of the laser. The RIN at the fiber-end output is also higher than the pump RIN from 30 kHz to 100 MHz, which denotes amplification of the noise transferred by the pump. Please note that the roll-off at 3 GHz is due to the detection system. A peak also can be seen around 7 kHz in the end-fiber output due to the residual Raman fiber laser pump. The end-fiber output of the fiber also displays a RIN reduction in the lower frequency range, caused by the triple interaction between the Raman pump and the two co-propagating generated signals, as shown in [19].

**Figure 4:**
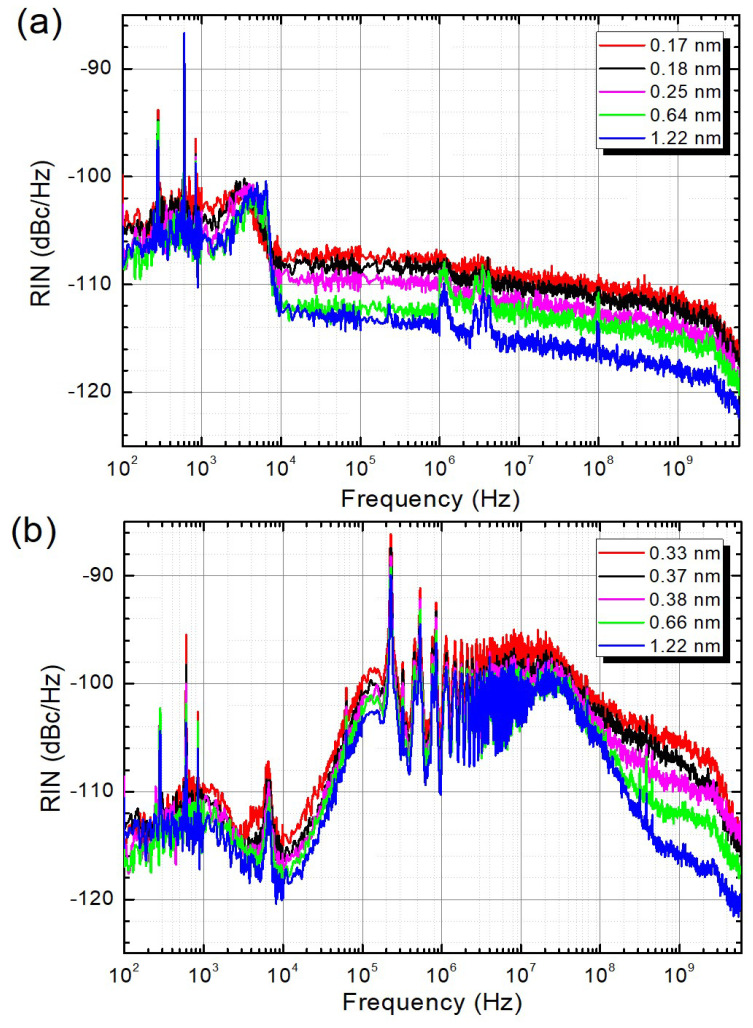
Relative intensity noise of the RDFL at the header (**a**) and end-fiber (**b**) outputs for different linewidths.

The correlation between the RIN and RDFL linewidth was studied by measuring the RIN for different bandwidths set in the programmable filter. Figure 4 shows the evolution of the RIN in both the header and end-fiber outputs for a variety of filter bandwidths. For this comparison, it is important to point out that each filter bandwidth generates a different laser linewidth for the header and end-fiber outputs. This effect is greater for narrow filters [7] and is also evidenced in Figure 2. As can be readily seen, the RIN of the laser increases for narrower linewidths.

**Figure 5:**
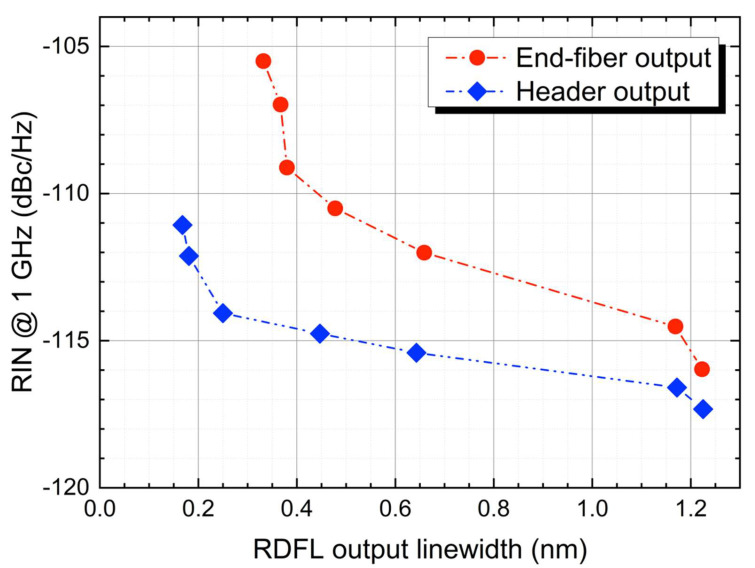
Evolution of the 50 Km RDFL RIN at 1 GHz, for the header (blue) and end-fiber (red) outputs.

When the linewidth of the laser is reduced, the total power is confined to a narrower band; therefore, its power spectral density is increased. This leads to a RIN increase that can be seen in Figure 4 for frequencies above 10 kHz for the header output and above 100 MHz in the case of the end-fiber output. A clearer way to see this effect is by comparing the evolution of noise transfer at a fixed frequency of 1 GHz (Figure 5), unaffected by the additional pump noise transfer, and before the roll-off due to the detection bandwidth.

**Figure 6:**
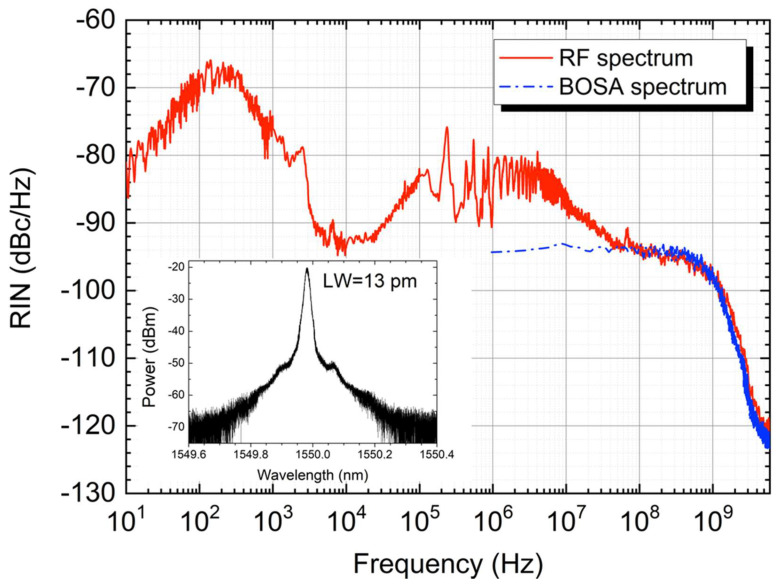
RIN measured at the end-fiber output for the PS-FBG configuration with a pump power of 2 W (red). Spectrum of the RDFL measured at the BOSA, at a resolution of 0.08 pm (inset). Superposition of the RDFL spectrum measured at the BOSA, converted to baseband (Blue).

Since the linewidth of the RDFL is broader than the bandwidth of the detection system, it is not possible with the initially proposed configuration to completely ascertain what dependence the RIN transfer function has on a further reduction in the laser spectral bandwidth, which should translate into a parallel filtering out of higher frequency signal modulation, and hence a drop in higher frequency RIN transfer. In order to verify this point, we modified the setup to generate a RDFL with a linewidth that fits into the detection bandwidth. A phase-shift fiber Bragg grating (PS-FBG) with a bandwidth of ∼7 pm was included after the programmable filter, as in [7]. Using this configuration, we achieved a narrow RDFL with a linewidth of only 13 pm (Figure 6 inset), measured using a Brillouin optical spectrum analyzer (BOSA OPT100 Aragon Photonics) with a resolution of 0.08 pm.

As shown in Figure 6, the RIN exhibits a roll-off around 800 MHz, corresponding to the half-linewidth (6.5 pm) of the laser, measured at the BOSA, confirming the expected impact on the RIN transfer function.

Please note that our results are fully consistent with previous observations [14,15] of increased laser spectral purity through the use of low-RIN pumping. More specifically, they represent evidence of the mirror phenomenon; that is, experimental demonstration of the possibility of reducing the output RIN in in RDFLs at high frequencies by reducing the bandwidth of the laser.

This effect can be easily understood by considering the nature of RIN, which effectively can be seen as a random amplitude modulation of the laser output. The minimum bandwidth of an amplitude modulated signal is given by twice the value of the modulation frequency. Hence, by reducing the spectral bandwidth, we are filtering out those oscillations with a modulation frequency higher than the half linewidth of our laser. This bandwidth limitation on RIN frequencies is not commonly observed in fiber lasers, since these sources generally present broad spectra, and thus the corresponding RIN cut-off frequency due to the laser linewidth is typically much higher than that imposed by the dispersion-induced walk-off between the pump and the signal.

The filtering effect is, on the other hand, clearly observable in our system: since the laser linewidth was reduced to 13 pm (approximately 1600 MHz), RIN transfer rolls off at precisely half this linewidth; that is, 800 MHz.

In other words, we can eliminate RIN transfer at high frequencies in RDFLs by acting directly on the output laser bandwidth through filtering techniques. This approach can be combined with the design considerations described in [19] to further reduce the overall impact of RIN transfer in RDFLs relying on standard, inherently noisy laser pumps.

## 4. Conclusions

The dependence of output RIN transfer on laser linewidth in ultralong RDFLs and the possibility of effecting control on the RIN intensity and its cut-off frequency through the use of spectral filtering were demonstrated for the first time. To achieve this, we have experimentally characterized the output RIN of a variable laser linewidth RDFL based on a single-arm, 50-km standard single-mode fiber, operating in the C-Band, with a forward-pumping topology, in which the wavelength and linewidth selection of the laser were adjusted by using a combination of programmable filter and a phase-shift grating filter in a loop mirror configuration.

For our lowest available filter bandwidth of 7 pm, a minimum RDFL output bandwidth of 13 pm was achieved, associated with a RIN cut-off frequency of 800 MHz. The obtained results open up new possibilities for the design of RIN-optimized RDFLs for specific applications with the appropriate choice of reflector bandwidth (e.g., through the use of an optimal fiber Bragg grating), or the implementation of flexible setups with variable filters.

## Data Availability

The data presented in this study are available upon reasonable request from the corresponding author.

## References

[1] Churkin D.V., Sugavanam S., Vatnik I.D., Wang Z., Podivilov E.V., Babin S.A., Rao Y., Turitsyn S.K. (2015). Recent Advances in Fundamentals and Applications of Random Fiber Lasers. Adv. Opt. Photonics.

[2] Wiersma D.S. (2008). The Physics and Applications of Random Lasers. Nat. Phys..

[3] Turitsyn S.K., Babin S.A., El-Taher A.E., Harper P., Churkin D.V., Kablukov S.I., Ania-Castañón J.D., Karalekas V., Podivilov E.V. (2010). Random Distributed Feedback Fibre Laser. Nat. Photonics.

[4] Rota-Rodrigo S., Gouhier B., Dixneuf C., Antoni-Micollier L., Guiraud G., Leandro D., Lopez-Amo M., Traynor N., Santarelli G. (2018). Watt-Level Green Random Laser at 532 Nm by SHG of a Yb-Doped Fiber Laser. Opt. Lett..

[5] Zhang L., Xu Y., Lu P., Mihailov S., Chen L., Bao X. (2018). Multi-Wavelength Brillouin Random Fiber Laser via Distributed Feedback From a Random Fiber Grating. J. Light. Technol..

[6] Wu H., Wang Z., He Q., Sun W., Rao Y. (2017). Common-Cavity Ytterbium/Raman Random Distributed Feedback Fiber Laser. Laser Phys. Lett..

[7] Leandro D., Rota-Rodrigo S., Ardanaz D., Lopez-Amo M. (2015). Narrow-Linewidth Multi-Wavelength Random Distributed Feedback Laser. J. Light. Technol..

[8] Du X., Zhang H., Wang X., Zhou P. (2015). Tunable Random Distributed Feedback Fiber Laser Operating at 1 Μm. Appl. Opt..

[9] Bravo M., Fernandez-Vallejo M., Lopez-Amo M. (2013). Internal Modulation of a Random Fiber Laser. Opt. Lett..

[10] DeMiguel-Soto V., Leandro D., Lopez-Amo M. (2018). Ultra-Long (290 Km) Remote Interrogation Sensor Network Based on a Random Distributed Feedback Fiber Laser. Opt. Express.

[11] Nuño J., Alcon-Camas M., Ania-Castañón J.D. (2012). RIN Transfer in Random Distributed Feedback Fiber Lasers. Opt. Express.

[12] Rizzelli G., Iqbal M.A., Gallazzi F., Rosa P., Tan M., Ania-Castañón J.D., Krzczanowicz L., Corredera P., Phillips I., Forysiak W. (2016). Impact of Input FBG Reflectivity and Forward Pump Power on RIN Transfer in Ultralong Raman Laser Amplifiers. Opt. Express.

[13] Han B., Rao Y., Wu H., Yao J., Guan H., Ma R., Wang Z. (2020). Low-Noise High-Order Raman Fiber Laser Pumped by Random Lasing. Opt. Lett..

[14] Ye J., Ma X., Zhang Y., Xu J., Zhang H., Yao T., Leng J., Zhou P. (2022). Revealing the Dynamics of Intensity Fluctuation Transfer in a Random Raman Fiber Laser. Photonics Res..

[15] Deheri R., Dash S., Supradeepa V.R., Balaswamy V. (2022). Cascaded Raman Fiber Lasers with Ultrahigh Spectral Purity. Opt. Lett..

[16] Han B., Dong S., Liu Y., Wang Z. (2022). Cascaded Random Raman Fiber Laser With Low RIN and Wide Wavelength Tunability. Photonic Sens..

[17] Nuño J., Ania-Castañón J.D. Anomalous RIN Transfer Function in Random Raman Lasers. Proceedings of the 2015 European Conference on Lasers and Electro-Optics—European Quantum Electronics Conference, paper CJ_P_21.

[18] Rota-Rodrigo S., Leandro D., Rizzelli G., Ania-Castañón J.D., Santarelli G., Lopez-Amo M. Experimental Observation of Anomalous RIN Transfer in Random Distributed Feedback Raman Fiber Lasers. Proceedings of the 2019 Conference on Lasers and Electro-Optics Europe and European Quantum Electronics Conference, paper cj_p_78.

[19] Rota-Rodrigo S., Rizzelli G., Leandro D., Nuño J., Lopez-Amo M., Santarelli G., Ania-Castañón J.D. (2020). Anomalous Relative Intensity Noise Transfer in Ultralong Random Fiber Lasers. Opt. Express.

[20] Babin S.A., El-Taher A.E., Harper P., Podivilov E.V., Turitsyn S.K. (2011). Tunable Random Fiber Laser. Phys. Rev. A.

[21] Babin S.A., Karalekas V., Podivilov E.V., Mezentsev V.K., Harper P., Ania-Castañón J.D., Turitsyn S.K. (2008). Turbulent Broadening of Optical Spectra in Ultralong Raman Fiber Lasers. Phys. Rev. A.

